# Supplementary material for: Low-Carbohydrate Diets and Mortality in Older Asian People: A 15-Year Follow-Up from a Prospective Cohort Study
Source: Nutrients. 2022 Mar 28;14(7):1406. doi: 10.3390/nu14071406 (PMC9002516; doi:10.3390/nu14071406)
Supplement: Supplementary file 1 [file nutrients-14-01406-s001.zip › nutrients-1604383-supplementary.pdf]

File Content

Table S1. Criteria for determining the low-carbohydrate-diet score

Table S2 Criteria for determining the low-carbohydrate-diet score in participants without and with diabetes

Table S3 Baseline characteristics by quartiles of low-carbohydrate-diet scores in 17,416 participants without diabetes and 2,790 participants with diabetes recruited in 2003-2008 and followed up till April 19, 2021

Table S4 Association of low-carbohydrate-diet (LCD) score with all-cause mortality in participants without and with diabetes

Table S5 Hazard ratios (HRs) of mortality from all-cause, cancer, CVD and other causes in low-carbohydrate score

Table S6 Hazard ratios (HRs) of mortality from all-cause, cancer, CVD and other causes in low-carbohydrate score of participants without and with diabetes

Table S7 Hazard ratios (HRs) of mortality from all-cause, cancer, CVD and other causes in low-carbohydrate score by excluding deaths during first three years follow-up

Table S8 Hazard ratios (HRs) of mortality from all-cause, cancer, CVD and other causes in low-carbohydrate score by excluding during first three years follow-up in participants without and with diabetes

Figure S1 Flowchart showing selection of participants included in the main analysis from Guangzhou Biobank Cohort Study

Figure S2 Association of overall LCD score with all-cause mortality by subgroup

Figure S3 Association of vegetable-based LCD score with all-cause mortality by subgroup

Figure S4 Association of meat-based LCD score with all-cause mortality by subgroup

Figure S5 Association of overall LCD score with all-cause mortality in by subgroup (A: participants without diabetes; B: participants with diabetes)

Figure S6 Association of vegetable-based LCD score with all-cause mortality by subgroup (A: participants without diabetes; B: participants with diabetes)

Figure S7 Association of meat-based LCD score with all-cause mortality by subgroup (A: participants without diabetes; B: participants with diabetes)

Table S9 A Guideline for Reporting Mediation Analyses Short-Form (AGReMA-SF) Checklist

Table S1. Criteria for determining the low-carbohydrate-diet score.

Point	Total carbohydrate	Low-quality carbohydrate	High-quality carbohydrate	Total Fat	Saturated fat	Unsaturated fat	Total protein	Animal protein	Plant protein
	Percentage of Energy								
0	> 68.8	> 60.7	> 21.2	< 17.4	< 2.5	< 6.0	<12.2	< 3.9	< 6.4
1	65.0-68.7	56.2-60.7	16.2-21.2	17.4-20.7	2.5-3.1	6.0-8.4	12.2-13.4	3.9-4.9	6.4-7.1
2	62.4-65.0	52.8-56.2	13.1-16.2	20.7-23.2	3.1-3.7	8.4-10.4	13.4-14.1	4.9-5.6	7.1-7.5
3	60.2-62.4	50.2-52.8	11.0-13.1	23.2-25.3	3.7-4.1	10.4-12.0	14.1-14.8	5.6-6.2	7.5-7.9
4	58.2-60.2	47.6-50.2	9.4-11.0	25.3-27.1	4.1-4.6	12.0-13.6	14.8-15.5	6.2-6.8	7.9-8.2
5	56.3-58.2	45.1-47.6	8.1-9.4	27.1-29.0	4.6-5.0	13.6-15.1	15.5-16.1	6.8-7.4	8.2-8.6
6	54.2-56.3	42.5-45.1	6.9-8.1	29.0-31.1	5.0-5.5	15.1-16.8	16.1-16.7	7.4-8.1	8.6-9.0
7	51.8-54.1	39.7-42.5	5.8-6.9	31.1-33.4	5.5-6.0	16.8-18.7	16.7-17.4	8.1-8.8	9.0-9.4
8	49.0-51.8	36.3-39.7	4.6-5.8	33.4-36.2	6.0-6.6	18.7-21.1	17.4-18.3	8.8-9.8	9.4-9.9
9	44.8-49.0	31.4-36.3	3.4-4.6	36.2-40.3	6.6-7.6	21.1-24.6	18.3-19.8	9.8-11.3	9.9-10.7
10	< 44.9	<31.4	< 3.4	>40.3	> 7.6	> 24.6	>19.8	> 11.3	> 10.7

High-quality carbohydrate was defined as carbohydrate from whole grains, whole fruit, legumes, and non-starchy vegetables.

Low-quality carbohydrate was defined as carbohydrate from refined grains, added sugar, fruit juice, potato, other starchy vegetables, and other sources.

Table S2. Criteria for determining the low-carbohydrate-diet score in participants without and with diabetes.

Point	Total carbohydrate	Low-quality carbohydrate	High-quality carbohydrate	Total Fat	Saturated fat	Unsaturated fat	Total protein	Animal protein	Plant protein
	Percentage of Energy								
**Participants without diabetes**									
0	> 68.8	> 60.9	> 21.0	< 17.4	< 2.4	< 5.9	<12.2	< 3.9	< 6.4
1	65.1-68.7	56.3-60.9	16.1-21.0	17.4-20.7	2.4-3.1	5.9-8.4	12.2-13.3	3.9-4.9	6.4-7.0
2	62.5-65.1	52.9-56.3	13.1-16.1	20.7-23.1	3.1-3.6	8.4-10.4	13.3-14.1	4.9-5.6	7.0-7.5
3	60.3-62.5	50.3-52.9	11.0-13.1	23.1-25.2	3.6-4.1	10.4-12.0	14.1-14.8	5.6-6.2	7.5-7.9
4	58.3-60.3	47.7-50.3	9.4-11.0	25.2-27.0	4.1-4.5	12.0-13.5	14.8-15.4	6.2-6.8	7.9-8.2
5	56.4-58.3	45.3-47.7	8.1-9.4	27.0-28.9	4.5-5.0	13.5-15.1	15.4-16.0	6.8-7.4	8.2-8.5
6	54.3-56.4	42.6-45.3	6.9-8.1	28.9-31.0	5.0-5.4	15.1-16.7	16.0-16.6	7.4-8.0	8.5-8.9
7	52.1-54.3	39.8-42.6	5.8-6.9	31.0-33.3	5.4-5.9	16.7-18.7	16.6-17.3	8.0-8.8	8.9-9.3
8	49.2-52.1	36.5-39.8	4.6-5.8	33.3-36.0	5.9-6.6	18.7-21.0	17.3-18.2	8.8-9.7	9.3-9.9
9	44.9-49.2	31.8-36.5	3.4-4.6	36.0-40.2	6.6-7.5	21.0-24.6	18.2-19.6	9.7-11.2	9.9-10.6
10	< 44.9	<31.8	< 3.4	>40.2	> 7.5	> 24.6	>19.6	> 11.2	> 10.6
**Participants with diabetes**									
0	> 68.3	> 60.1	> 22.4	< 17.5	< 2.6	< 6.0	<12.5	< 4.0	< 6.5
1	64.4-68.3	55.5-60.1	16.6-22.4	17.5-21.0	2.6-3.3	6.0-8.6	12.5-13.7	4.0-5.0	6.5-7.2
2	61.7-64.4	52.3-55.5	13.3-16.6	21.0-23.7	3.3-3.8	8.6-10.4	13.7-14.5	5.0-5.7	7.2-7.6
3	59.3-61.7	49.4-52.3	11.1-13.3	23.7-25.7	3.8-4.3	10.4-12.0	14.5-15.2	5.7-6.4	7.6-8.0
4	57.4-59.3	46.8-49.4	9.4-11.1	25.7-27.8	4.3-4.7	12.0-13.6	15.2-15.9	6.4-7.0	8.0-8.5
5	55.1-57.4	44.2-46.8	8.0-9.4	27.8-29.9	4.5-5.2	13.6-15.3	15.9-16.6	7.0-7.6	8.5-8.9
6	52.9-55.1	41.2-44.2	6.9-8.0	29.9-31.9	5.2-5.7	15.3-17.0	16.6-17.3	7.6-8.4	8.9-9.3
7	50.5-52.9	38.3-41.2	5.8-6.9	31.9-34.3	5.7-6.2	17.0-19.0	17.3-18.1	8.4-9.2	9.3-9.7
8	47.7-50.5	34.9-38.3	4.6-5.8	34.3-37.1	6.2-6.9	19.0-21.5	18.1-19.1	9.2-10.3	9.7-10.3
9	43.8-47.7	29.3-34.9	3.3-4.6	37.1-41.1	6.9-8.0	21.5-24.9	19.1-20.6	10.3-11.7	10.3-11.2
10	< 43.8	<29.3	< 3.3	>41.1	> 8.0	> 24.9	>20.6	> 11.7	> 11.2

High-quality carbohydrate was defined as carbohydrate from whole grains, whole fruit, legumes, and non-starchy vegetables.

Low-quality carbohydrate was defined as carbohydrate from refined grains, added sugar, fruit juice, potato, other starchy vegetables, and other sources.

Table S3. Baseline characteristics by quartiles of low-carbohydrate-diet scores in 17,416 participants without diabetes and 2,790 participants with diabetes recruited in 2003-2008 and followed up till April 19, 2021 [1.]

Characteristic	Overall low- carbohydrate -diet score		*P*-value	Vegetable-based low-carbohydrate-diet score		*P*-value	Meat-based low-carbohydrate-diet score		*P*-value
	Quartile 1	Quartile 4		Quartile 1	Quartile 4		Quartile 1	Quartile 4	
**Participants without diabetes**									
Number of participants	4,507	4,086		4,669	3,146		4,432	3,807	
Age, mean (SD), years	63.3 (6.5)	61.8 (6.8)	<0.001	62.5 (6.6)	62.6 (6.7)	0.19	63.3 (6.6)	61.7 (6.9)	0.02
Sex			<0.001			0.76			<0.001
Women	2,971 (65.9)	3,070 (73.1)		3,305 (70.8)	2,220 (70.6)		2,935 (64.8)	2,859 (77.8)	
Men	1,536 (34.1)	1,016 (26.9)		1,364 (29.2)	962 (29.4)		1,593 (35.2)	809 (22.2)	
Education level			<0.001			<0.001			<0.001
Less than primary school	2,315 (51.4)	1,642 (40.2)		1,941 (41.6)	1,559 (49.5)		2,435 (53.8)	1,310 (35.7)	
Middle school	1,810 (40.2)	2,043 (50.0)		2,215 (47.5)	1,343 (42.7)		1,787 (39.5)	1,958 (53.4)	
College or above	379 (8.4)	401 (9.8)		511 (10.9)	244 (7.8)		303 (6.7)	309 (10.9)	
Family income, RMB/year			<0.001			0.22			<0.001
<20,000	1,100 (24.4)	680 (16.7)		855 (18.3)	623 (19.9)		1,112 (24.6)	570 (15.6)	
20,000-30,000	873 (19.4)	750 (18.4)		943 (20.2)	606 (19.3)		876 (19.4)	676 (18.5)	
30,000-50,000	703 (15.6)	889 (21.8)		886 (19.0)	592 (18.9)		729 (16.1)	800 (21.8)	
≥ 50,000	417 (9.3)	765 (18.8)		687 (14.7)	473 (15.1)		397 (8.8)	761 (20.8)	
Do not know	1,362 (31.2)	995 (24.4)		1,291 (27.7)	844 (26.9)		1,406 (31.1)	855 (23.3)	
Smoking			<0.001			<0.001			<0.001
Never	3,468 (77.0)	3,347 (82.0)		3,811 (81.6)	2,398 (76.3)		3,405 (75.3)	3,098 (84.5)	
Ever	500 (11.1)	305 (7.5)		461 (9.9)	314 (9.6)		517 (11.4)	258 (7.0)	
Current	537 (11.9)	430 (10.5)		396 (8.5)	442 (14.1)		603 (13.3)	309 (8.4)	
Drinking			<0.001			0.78			0.002
Never	3,650 (81.4)	3,228 (79.2)		3,674 (79.1)	2,518 (80.2)		3,642 (81.8)	2,929 (80.2)	
Ever	112 (2.5)	74 (1.8)		91 (2.0)	67 (2.1)		110 (2.4)	57 (1.6)	
Current	721 (16.1)	773 (19.0)		879 (18.9)	554 (17.7)		755 (16.8)	667 (18.3)	
Physical activity			<0.001			<0.001			<0.001

Low	279 (6.2)	426 (10.4)		262 (5.6)	439 (14.0)		341 (7.5)	355 (9.7)	
Moderate	1,837 (40.8)	2,156 (52.8)		2,139 (45.8)	1,483 (47.1)		1,853 (40.9)	1,926 (52.5)	
High	2,391 (53.1)	1,504 (36.8)		2,268 (48.5)	1,224 (38.9)		2,334 (51.6)	1,387 (37.8)	
BMI, mean (SD), kg/m^2	23.7 (3.3)	23.5 (3.3)	0.10	23.6 (3.2)	23.6 (3.3)	0.92	23.7 (3.3)	23.6 (3.3)	0.01

Continued Table S3

Characteristic	Overall low- carbohydrate -diet score		*P*-value	Vegetable-based low-carbohydrate-diet score		*P*-value	Meat-based low-carbohydrate-diet score		*P*-value
	Quartile 1	Quartile 4		Quartile 1	Quartile 4		Quartile 1	Quartile 4	
Dietary intake, mean (SD)									
Total energy, kcal/d	1,862 (523)	1,731 (503)	<0.001	1,820 (529)	1,794 (488)	<0.001	1,842 (509)	1,789 (524)	<0.001
Total carbohydrate, percent of total energy intake	67.5 (4.7)	46.6 (5.4)	<0.001	60.9 (8.0)	52.5 (8.7)	<0.001	66.0 (6.0)	48.3 (7.4)	<0.001
High-quality carbohydrate	10.0 (7.2)	9.9 (6.0)	<0.001	14.9 (7.1)	5.6 (3.7)	<0.001	7.4 (4.4)	13.7 (8.4)	<0.001
Low-quality carbohydrate	57.4 (8.9)	36.5 (6.9)	<0.001	45.8 (10.1)	46.8 (9.7)	<0.001	58.6 (6.6)	34.4 (6.9)	<0.001
Total protein, percent of total energy intake	14.2 (1.9)	17.9 (2.8)	<0.001	16.4 (2.9)	15.1 (3.0)	<0.001	13.9 (2.0)	18.1 (2.8)	<0.001
Animal protein	5.2 (1.8)	10.0 (2.9)	<0.001	8.0 (3.1)	6.7 (2.8)	<0.001	5.1 (1.7)	10.0 (2.9)	<0.001
Plant protein	9.0 (1.3)	7.9 (1.7)	<0.001	8.4 (1.6)	8.5 (1.7)	<0.001	8.8 (1.3)	8.1 (1.9)	<0.001
Total fat, percent of total energy intake	12.8 (5.6)	26.4 (7.4)	<0.001	14.3 (5.9)	27.1 (7.8)	<0.001	13.7 (6.3)	27.2 (7.6)	<0.001
Saturated fat	3.4 (1.5)	6.2 (1.7)	<0.001	4.3 (1.7)	5.5 (1.7)	<0.001	3.0 (1.0)	7.0 (1.4)	<0.001
Monounsaturated fat	5.5 (2.5)	11.4 (3.4)	<0.001	6.2 (2.7)	11.4 (3.7)	<0.001	5.8 (2.7)	11.4 (3.7)	<0.001
Polyunsaturated fat	3.9 (2.4)	8.4 (3.4)	<0.001	4.1 (2.5)	9.1 (3.7)	<0.001	4.3 (2.8)	8.1 (3.6)	<0.001
History of CVD	1,711 (38.3)	1,586 (39.3)	0.25	1,833 (39.8)	1,193 (38.4)	0.41	1,660 (37.0)	1,491 (41.2)	0.001
History of cancer	86 (1.9)	81 (2.0)	0.97	118 (2.6)	39 (1.3)	0.001	90 (2.0)	81 (2.2)	<0.001
Fasting plasma-glucose, mmol/L	5.4 (0.6)	5.2 (0.6)	<0.001	5.3 (0.6)	5.2 (0.6)	<0.001	5.4 (0.6)	5.2 (0.6)	<0.001
Systolic blood pressure, mmHg	132.5 (22.3)	128.5 (22.1)	<0.001	129.3 (21.9)	131.6 (22.4)	<0.001	132.5 (22.2)	128.0 (21.9)	<0.001
Total cholesterol, mmol/L	3.7 (1.0)	3.7 (1.0)	0.43	3.7 (1.0)	3.6 (0.9)	<0.001	3.7 (1.0)	3.7 (1.0)	0.58

Self-rated health									
Good/very good	3,843 (86.1)	3,431 (85.1)	0.04	3,979 (86.4)	2,643 (85.2)	0.03	3,863 (86.2)	3,065 (84.7)	0.02
Poor/very poor	619 (13.9)	602 (14.9)		629 (13.6)	460 (14.8)		619 (13.8)	553 (15.3)	
**Participants with diabetes**									
Number of participants	701	696		736	526		709	619	
Age, mean (SD), year	64.5 (6.3)	63.7 (6.2)	0.64	64.6 (6.3)	63.9 (6.0)	0.70	64.3 (6.3)	63.8 (6.2)	0.80
Sex			0.69			0.15			0.03
Women	516 (73.6)	524 (75.3)		533 (72.4)	378 (71.9)		511 (72.1)	458 (78.4)	
Men	185 (26.4)	172 (24.7)		203 (27.6)	148 (28.1)		198 (27.9)	134 (21.6)	

Continued Table S3

Characteristic	Overall low- carbohydrate -diet score		*P*-value	Vegetable-based low-carbohydrate-diet score		*P*-value	Meat-based low-carbohydrate-diet score		*P*-value
	Quartile 1	Quartile 4		Quartile 1	Quartile 4		Quartile 1	Quartile 4	
Education level			<0.001			0.03			<0.001
Less than primary school	414 (59.2)	290 (41.7)		361 (49.1)	266 (50.6)		430 (60.8)	250 (40.4)	
Middle school	242 (34.6)	329 (47.3)		291 (39.5)	222 (42.2)		242 (34.2)	291 (47.0)	
College or above	43 (6.2)	77 (11.1)		84 (11.4)	38 (7.2)		35 (5.0)	78 (12.6)	
Family income, RMB/year			<0.001			0.24			<0.001
<20,000	187 (26.7)	116 (16.7)		162 (22.0)	123 (23.5)		202 (28.5)	94 (15.2)	
20,000-30,000	130 (18.6)	152 (21.9)		148 (20.1)	116 (22.1)		141 (19.9)	136 (22.0)	
30,000-50,000	93 (13.3)	142 (20.4)		112 (15.2)	78 (14.9)		93 (13.1)	121 (19.6)	
≥ 50,000	67 (9.6)	112 (16.1)		100 (13.6)	64 (12.2)		55 (7.8)	100 (16.2)	
Do not know	223 (31.9)	173 (24.9)		214 (29.1)	143 (27.3)		21 (30.7)	168 (27.1)	
Smoking			0.72			0.06			0.04
Never	568 (81.0)	566 (81.6)		597 (81.1)	408 (77.9)		565 (79.7)	529 (85.6)	
Ever	88 (12.6)	72 (10.4)		87 (11.8)	62 (11.8)		91 (12.8)	51 (8.3)	
Current	45 (6.4)	56 (8.1)		52 (7.1)	54 (10.3)		53 (7.5)	38 (6.2)	
Drinking			0.94			0.15			0.03

Never	576 (82.2)	558 (80.5)		592 (80.7)	448 (85.8)		572 (80.7)	511 (82.8)	
Ever	18 (2.6)	19 (2.7)		18 (2.5)	10 (1.9)		18 (2.5)	21 (3.4)	
Current	107 (15.3)	116 (16.7)		124 (16.9)	64 (12.3)		119 (16.8)	85 (13.8)	
Physical activity			<0.001			<0.001			0.005
Low	34 (4.9)	44 (6.3)		26 (3.5)	52 (9.9)		43 (6.1)	36 (5.8)	
Moderate	319 (45.5)	383 (55.0)		350 (47.6)	276 (52.5)		336 (47.4)	351 (56.7)	
High	348 (49.6)	269 (38.7)		360 (48.9)	198 (37.6)		330 (46.5)	232 (37.5)	
BMI, mean (SD), kg/m^2	25.0 (3.1)	24.5 (3.1)	0.16	24.7 (3.1)	24.6 (3.0)	0.07	25.1 (3.3)	24.3 (3.1)	0.64
Dietary intake, mean (SD)									
Total energy, kcal/d	1,804 (506)	1,705 (507)	<0.001	1,800 (526)	1,729 (479)	<0.001	1,785 ((490)	1,738 (492)	<0.001
Total carbohydrate, percent of total energy intake	67.1 (4.8)	45.3 (5.3)	<0.001	60.6 (8.4)	50.9 (8.3)	<0.001	65.3 (6.3)	46.9 (7.4)	<0.001
High-quality carbohydrate	10.4 (7.6)	10.5 (6.9)	<0.001	14.9 (7.7)	5.5 (3.8)	<0.001	7.4 (4.3)	14.6 (9.4)	<0.001
Low-quality carbohydrate	56.6 (8.9)	34.6 (7.4)	<0.001	45.6 (11.1)	45.2 (9.3)	<0.001	57.7 (6.5)	32.1 (7.3)	<0.001

Continued Table S3

Characteristic	Overall low- carbohydrate -diet score		*P*-value	Vegetable-based low-carbohydrate-diet score		*P*-value	Meat-based low-carbohydrate-diet score		*P*-value
	Quartile 1	Quartile 4		Quartile 1	Quartile 4		Quartile 1	Quartile 4	
Total protein, percent of total energy intake	14.5 (2.0)	18.7 (2.9)	<0.001	16.9 (3.1)	15.7 (3.2)	<0.001	14.2 (2.1)	19.0 (3.0)	<0.001
Animal protein	5.3 (2.0)	10.4 (2.9)	<0.001	8.2 (3.2)	7.0 (2.8)	<0.001	5.2 (1.8)	10.4 (2.9)	<0.001
Plant protein	9.2 (1.4)	8.4 (2.1)	<0.001	8.7 (1.7)	8.7 (2.1)	<0.001	9.0 (1.4)	8.6 (2.2)	<0.001
Total fat, percent of total energy intake	19.9 (5.1)	37.9 (6.1)	<0.001	24.2 (7.4)	34.9 (8.0)	<0.001	22.1 (6.8)	36.0 (8.2)	<0.001
Saturated fat	3.5 (1.6)	6.4 (1.9)	<0.001	4.4 (1.9)	5.8 (1.8)	<0.001	3.1 (1.1)	7.4 (1.6)	<0.001
Monounsaturated fat	5.5 (2.4)	11.3 (3.5)	<0.001	6.0 (2.6)	11.6 (3.7)	<0.001	5.9 (2.8)	11.4 (3.8)	<0.001
Polyunsaturated fat	3.9 (2.5)	8.6 (3.6)	<0.001	3.9 (2.4)	9.7 (3.9)	<0.001	4.6 (3.1)	8.5 (3.9)	<0.001
History of CVD	408 (41.0)	378 (54.8)	0.18	410 (56.6)	312 (59.5)	0.99	393 (56.4)	337 (55.0)	0.06

History of cancer	13 (1.9)	19 (2.8)	0.14	21 (2.9)	14 (2.7)	0.76	11 (1.6)	18 (2.9)	0.04
Fasting plasma-glucose, mmol/L	8.6 (3.1)	8.7 (3.1)	0.37	8.9 (3.0)	8.5 (3.3)	0.01	8.6 (3.2)	8.6 (3.0)	0.09
Systolic blood pressure, mmHg	140.4 (22.7)	137.3 (21.6)	0.04	138.5 (22.4)	137.9 (20.9)	0.93	140.2 (22.7)	137.3 (21.6)	0.02
Total cholesterol, mmol/L	3.9 (1.1)	3.9 (1.1)	0.93	4.0 (1.1)	3.8 (1.1)	0.04	3.8 (1.1)	3.8 (1.0)	0.48
Self-rated health			0.005			0.04			0.04
Good/very good	453 (77.3)	618 (73.3)		471 (78.5)	453 (72.5)		490 (78.4)	539 (72.2)	
Poor/very poor	133 (22.7)	225 (26.7)		129 (21.5)	172 (27.5)		135 (21.6)	207 (27.8)	

Abbreviations: BMI, body mass index; CVD, Cerebrovascular disease; SD, Standard Deviation;

[1] Data are presented as number (percentage) of study participants unless otherwise indicated.

Note: one dollar almost equals to 7 RMB.

Table S4. Association of low-carbohydrate-diet (LCD) score with all-cause mortality in participants without and with diabetes.

Characteristic	Quartiles of LCD scores				*P* for trend	*P* for non-linear
	1	2	3	4		
**Participants without diabetes**						
Overall LCD score [a]						
Median score (IQR)	6 (4, 8)	13 (11, 14)	18 (17, 19)	24 (22, 26)		
Person-years of follow-up	66,071	68,119	61,773	59,855		
Mortality rate (per 1000 person-years)	171.0	147.1	124.2	127.3		
Crude HR (95% CI)	1.00	0.87 (0.80-0.95) *	0.74 (0.68-0.81) *	0.76 (0.70-0.84) *	<0.001	0.12
Adjusted HR (95% CI) [d]	1.00	0.92 (0.85-1.00)	0.86 (0.78-0.94) *	0.90 (0.82-0.99) *	0.005	0.06
Adjusted HR (95% CI) [e]	1.00	0.94 (0.86-1.03)	0.88 (0.80-0.98) *	0.92 (0.83-1.01)	0.03	0.19
Adjusted HR (95% CI) [f]	1.00	0.96 (0.87-1.04)	0.90 (0.81-0.99) *	0.93 (0.84-1.03)	0.08	0.25
Vegetable-based LCD score [b]						

Median score (IQR)	11 (9, 12)	14 (13, 15)	17 (16.18)	20 (19, 21)		
Person-years of follow-up	68,770	74,652	66,646	45,749		
Mortality rate (per 1000 person-years)	140.5	135.3	146.7	154.5		
Crude HR (95% CI)	1.00	0.97 (0.89-1.06)	1.06 (0.97-1.15)	1.12 (1.02-1.23) *	0.008	0.15
Adjusted HR (95% CI) [d]	1.00	0.97 (0.89-1.06)	1.09 (0.99-1.19)	1.15 (1.04-1.26) *	0.001	0.16
Adjusted HR (95% CI) [e]	1.00	0.97 (0.88-1.06)	1.06 (0.97-1.17)	1.10 (1.01-1.23) *	0.02	0.26
Adjusted HR (95% CI) [f]	1.00	0.96 (0.88-1.05)	1.04 (0.95-1.15)	1.06 (0.96-1.18)	0.12	0.30
Meat-based LCD score [c]						
Median score (IQR)	6 (3, 8)	13 (11, 14)	19 (17, 20)	24 (23, 26)		
Person-years of follow-up	66,057	66,297	69,618	53,845		
Mortality rate (per 1000 person-years)	175.9	145.3	128.0	119.8		
Crude HR (95% CI)	1.00	0.83 (0.76-0.90) **	0.74 (0.68-0.81) ***	0.70 (0.63-0.77) ***	<0.001	0.06
Adjusted HR (95% CI) [d]	1.00	0.89 (0.81-0.97) *	0.84 (0.77-0.92) **	0.83 (0.75-0.91) **	<0.001	0.13
Adjusted HR (95% CI) [e]	1.00	0.91 (0.83-0.99) *	0.89 (0.82-0.98) *	0.87 (0.79 o 0.97) **	0.006	0.28
Adjusted HR (95% CI) [f]	1.00	0.91 (0.83-0.99) *	0.91 (0.83-1.00) *	0.90 (0.82-1.00) *	0.02	0.26

Continued Table S4

Characteristic	Quartiles of LCD scores 1	2	3	4	*P* for trend	*P* for non-linear
**Participants with diabetes**						
Overall LCD score [a]						
Median score (IQR)	6 (3, 8)	13 (11, 14)	18 (17, 19)	24 (22, 26)		
Person-years of follow-up	8,124	9,605	9,516	11,784		
Mortality rate (per 1000 person-years)	272.0	221.8	243.8	252.0		
Crude HR (95% CI)	1.00	0.81 (0.67-0.98)	0.91 (0.76-1.09)	0.94 (0.79-1.12)	0.97	0.06
Adjusted HR (95% CI) [d]	1.00	0.87 (0.72-1.05)	0.93 (0.78-1.12)	1.00 (0.84-1.19)	0.68	0.11

Adjusted HR (95% CI) [e]	1.00	0.91 (0.75-1.10)	0.95 (0.78-1.16)	1.09 (0.90-1.31)	0.26	0.09
Adjusted HR (95% CI) [f]	1.00	0.90 (0.74-1.09)	0.96 (0.79-1.17)	1.06 (0.88-1.28)	0.34	0.14
Vegetable-based LCD score [b]						
Median score (IQR)	11 (9, 12)	14 (13, 15)	17 (16, 18)	20 (19, 21)		
Person-years of follow-up	8,587	10,932	10,872	8,639		
Mortality rate (per 1000 person-years)	235.2	236.0	253.9	262.8		
Crude HR (95% CI)	1.00	1.00 (0.84-1.21)	1.10 (0.92-1.32)	1.15 (0.95-1.39)	0.08	0.78
Adjusted HR (95% CI) [d]	1.00	1.02 (0.85-1.22)	1.20 (1.00-1.44)	1.20 (1.00-1.45)	0.02	0.86
Adjusted HR (95% CI) [e]	1.00	1.03 (0.86-1.25)	1.21 (1.00-1.47)	1.18 (0.95-1.45)	0.04	0.46
Adjusted HR (95% CI) [f]	1.00	1.04 (0.86-1.26)	1.26 (1.00-1.53)	1.21 (0.98-1.49)	0.03	0.31
Meat-based LCD score [c]						
Median score (IQR)	6 (3, 8)	13 (11, 14)	18 (17, 20)	24 (23, 27)		
Person-years of follow-up	8,758	9,541	10,318	10,413		
Mortality rate (per 1000 person-years)	269.5	228.5	246.2	244.9		
Crude HR (95% CI)	1.00	0.85 (0.70-1.02)	0.93 (0.78-1.11)	0.93 (0.78-1.11)	0.66	0.21
Adjusted HR (95% CI) [d]	1.00	0.85 (0.71-1.03)	0.91 (0.76-1.09)	0.94 (0.79-1.12)	0.70	0.16
Adjusted HR (95% CI) [e]	1.00	0.87 (0.72-1.04)	0.94 (0.78-1.13)	0.99 (0.82-1.19)	0.85	0.16
Adjusted HR (95% CI) [f]	1.00	0.82 (0.67-1.00)	0.94 (0.78-1.13)	0.97 (0.80-1.17)	0.82	0.10

Abbreviations: IQR, Interquartile Range

[a] Low carbohydrate, high total fat, and high protein intake

[b] Low high-quality carbohydrate, high unsaturated fat, and high plant protein intake

[c] Low low-quality carbohydrate, high saturated fat, and high animal protein intake

[d]: Adjusted for sex and age

[e]: Additionally adjusted for education, family income, smoking, drinking, physical activity, BMI, and history of cancer and CVD.

[f] Additionally adjusted for systolic blood pressure, fasting plasma-glucose, total cholesterol and self-rated health at baseline.

[*]: 0.05; [**]: 0.01; [***]: 0.001

Table S5. Association of LCD score with mortality of cancer, CVD and other causes [1].

Characteristic	Quartiles of LCD scores				*P* for trend	*P* for non-linear
	1	2	3	4		
Overall low-carbohydrate-diet score [a]						
Cancer mortality	1.00	1.11 (0.96 to 1.28)	1.05 (0.90 to 1.23)	1.09 (0.90 to 1.23)	0.39	0.47
CVD mortality	1.00	0.85 (0.78 to 1.00)	0.83 (0.72 to 0.96) *	0.99 (0.87 to 1.14)	0.04	0.08
Other mortality	1.00	0.85 (0.73 to 1.00)	0.87 (0.74 to 1.03)	0.84 (0.71 to 1.00)	0.04	0.24
Vegetable-based low-carbohydrate-diet score [b]						
Cancer mortality	1.00	0.88 (0.77 to 1.02)	0.93 (0.80 to 1.08)	1.04 (0.89 to 1.23)	0.60	0.02
CVD mortality	1.00	1.18 (1.03 to 1.34) *	1.36 (1.18 to 1.56) ***	1.39 (1.19 to 1.62) ***	<0.001	0.15
Other mortality	1.00	0.89 (0.77 to 1.04)	1.06 (0.91 to 1.24)	1.03 (0.87 to 1.23)	0.31	0.38
Meat-based low-carbohydrate-diet score [c]						
Cancer mortality	1.00	0.99 (0.86 to 1.15)	1.16 (0.98 to 1.34)	1.05 (0.90 to 1.23)	0.12	0.48
CVD mortality	1.00	0.84 (0.75 to 0.95) **	0.82 (0.72 to 0.93) **	0.81 (0.70 to 0.93) *	0.02	0.10
Other mortality	1.00	0.95 (0.82 to 1.09)	0.82 (0.70 to 0.95) *	0.83 (0.70 to 0.98) *	0.04	0.26

[a] Low carbohydrate, high total fat, and high protein intake

[b] Low high-quality carbohydrate, high unsaturated fat, and high plant protein intake

[c] Low low-quality carbohydrate, high saturated fat, and high animal protein intake

[1] All the models adjusted for sex, age, education, family income, smoking, drinking, physical activity, BMI, and history of cancer and CVD.

*:<0.05; **<0.01; ***:<0.001

Table S6. Association of LCD score with mortality of cancer, CVD and other causes in participants without and with diabetes [1.]

Characteristic	Quartiles of LCD scores				*P* for trend	*P* for non-linear
	1	2	3	4		
**Participants without diabetes**						
Overall low-carbohydrate-diet score [a]						
Cancer mortality	1.00	1.13 (0.97 to 1.32)	1.08 (0.92 to 1.28)	1.08 (0.91 to 1.27)	0.50	0.23
CVD mortality	1.00	0.84 (0.77 to 1.02)	0.76 (0.64 to 0.89) **	0.88 (0.75 to 1.03)	0.04	0.06
Other mortality	1.00	0.83 (0.70 to 0.97) *	0.85 (0.71 to 1.02)	0.79 (0.66 to 1.00)	0.02	0.26
Vegetable-based low-carbohydrate-diet score [b]						
Cancer mortality	1.00	0.91 (0.78 to 1.06)	0.94 (0.80 to 1.10)	1.08 (0.90 to 1.29)	0.46	0.04
CVD mortality	1.00	1.16 (1.00 to 1.34)	1.27 (1.08 to 1.48) **	1.26 (1.06 to 1.51) *	0.003	0.19
Other mortality	1.00	0.83 (0.70 to 0.98) *	1.00 (0.84 to 1.19)	0.96 (0.78 to 1.17)	0.87	0.19
Meat-based low-carbohydrate-diet score [c]						
Cancer mortality	1.00	1.03 (0.88 to 1.20)	1.19 (1.02 to 1.39) *	1.06 (0.89 to 1.26)	0.18	0.22
CVD mortality	1.00	0.82 (0.71 to 0.94) **	0.76 (0.65 to 0.88) ***	0.79 (0.67 to 0.93) **	0.001	0.13
Other mortality	1.00	0.92 (0.79 to 1.09)	0.78 (0.66 to 0.93) *	0.80 (0.65 to 0.97) *	0.003	0.53
**Participants with diabetes**						
Overall low-carbohydrate-diet score [a]						
Cancer mortality	1.00	0.98 (0.68 to 1.41)	0.84 (0.57 to 1.23)	0.99 (0.69 to 1.43)	0.18	0.44
CVD mortality	1.00	0.81 (0.60 to 1.09)	0.94 (0.70 to 1.27)	1.15 (0.86 to 1.52)	0.01	0.06
Other mortality	1.00	1.00 (0.70 to 1.43)	1.08 (0.76 to 1.55)	1.09 (0.77 to 1.55)	0.57	0.99
Vegetable-based low-carbohydrate-diet score [b]						
Cancer mortality	1.00	0.75 (0.53 to 1.06)	0.82 (0.57 to 1.18)	0.82 (0.56 to 1.22)	0.44	0.25
CVD mortality	1.00	1.21 (0.89 to 1.63)	1.59 (1.18 to 2.15) **	1.54 (1.11 to 2.13) *	0.003	0.25
Other mortality	1.00	1.16 (0.82 to 1.64)	1.23 (0.86 to 1.76)	1.16 (0.79 to 1.70)	0.44	0.38
Meat-based low-carbohydrate-diet score [c]						
Cancer mortality	1.00	0.80 (0.55 to 1.16)	0.96 (0.68 to 1.38)	0.98 (0.69 to 1.41)	0.77	0.39
CVD mortality	1.00	0.79 (0.59 to 1.05)	0.94 (0.72 to 1.24)	0.94 (0.70 to 1.24)	0.97	0.28
Other mortality	1.00	1.04 (0.74 to 1.46)	0.91 (0.64 to 1.29)	1.05 (0.74 to 1.48)	0.97	0.66

[a] Low carbohydrate, high total fat, and high protein intake

[b] Low high-quality carbohydrate, high unsaturated fat, and high plant protein intake

[c] Low low-quality carbohydrate, high saturated fat, and high animal protein intake

[r] All the models adjusted for sex, age, education, family income, smoking, drinking, physical activity, BMI, and history of cancer and CVD.

[*]:<0.05; [**]<0.01; [***]:<0.001

[*]:<0.05; [**]<0.01; [***]:<0.001

Table S7. Association of LCD score with mortality of all-cause, cancer, CVD and other causes by excluding deaths during first three years follow-up [1].

Characteristic	Quartiles of LCD scores				*P* for trend
	1	2	3	4	
Overall low- carbohydrate -diet score [a]					
All-cause mortality	1.00	0.94 (0.87 to 1.02)	0.92 (0.85 to 1.01)	1.00 (0.92 to 1.09)	0.81
Cancer mortality	1.00	1.11 (0.97 to 1.28)	1.07 (0.91 to 1.25)	1.12 (0.96 to 1.31)	0.23
CVD mortality	1.00	0.88 (0.78 to 1.00)	0.83 (0.72 to 0.96) *	0.99 (0.86 to 1.14)	0.65
Other mortality	1.00	0.86 (0.74 to 1.00)	0.91 (0.77 to 1.07)	0.89 (0.76 to 1.05)	0.24
Vegetable low-carbohydrate-diet score [b]					
All-cause mortality	1.00	0.98 (0.91 to 1.07)	1.12 (1.03 to 1.22) *	1.15 (1.05 to 1.27) **	<0.001
Cancer mortality	1.00	0.88 (0.76 to 1.01)	0.94 (0.81 to 1.08)	1.04 (0.88 to 1.22)	0.60
CVD mortality	1.00	1.17 (1.02 to 1.34) *	1.35 (1.18 to 1.55) ***	1.38 (1.18 to 1.61) ***	<0.001
Other mortality	1.00	0.89 (0.76 to 1.04)	1.07 (0.91 to 1.25)	1.04 (0.87 to 1.24)	0.29
Meat-based low-carbohydrate-diet score [c]					
All-cause mortality	1.00	0.89 (0.82 to 0.97) *	0.90 (0.82 to 0.97) *	0.88 (0.81 to 0.96) *	0.008
Cancer mortality	1.00	1.00 (0.87 to 1.16)	1.17 (0.99 to 1.35)	1.09 (0.94 to 1.28)	0.07
CVD mortality	1.00	0.82 (0.73 to 0.93) **	0.82 (0.72 to 0.94) **	0.86 (0.75 to 0.98) *	0.01
Other mortality	1.00	0.94 (0.81 to 1.09)	0.81 (0.69 to 0.95) *	0.89 (0.75 to 1.05)	0.04

[a] Low carbohydrate, high total fat, and high protein intake

[b] Low high-quality carbohydrate, high unsaturated fat, and high plant protein intake

[c] Low low-quality carbohydrate, high saturated fat, and high animal protein intake

[1] All the models adjusted for sex, age, education, family income, smoking, drinking, physical activity, BMI, and history of cancer and CVD.

*:<0.05; **<0.01; ***:<0.001

Table S8. Association of LCD score with mortality of all-cause, cancer, CVD and other causes by excluding during first three years follow-up in participants without and with diabetes [1].

Characteristic	Quartiles of LCD scores				*P* for trend
	1	2	3	4	
**Participants without diabetes**					
Overall low- carbohydrate -diet score [a]					
All-cause mortality	1.00	0.95 (0.87 to 1.03)	0.89 (0.81 to 0.98) *	0.92 (0.83 to 1.02)	0.04
Cancer mortality	1.00	1.14 (0.98 to 1.33)	1.10 (0.93 to 1.30)	1.10 (0.93 to 1.31)	0.34
CVD mortality	1.00	0.88 (0.77 to 1.02)	0.75 (0.64 to 0.89) **	0.87 (0.74 to 1.02)	0.03
Other mortality	1.00	0.82 (0.70 to 0.97) *	0.84 (0.70 to 1.01)	0.80 (0.66 to 0.96) *	0.02
Vegetable low-carbohydrate-diet score [b]					
All-cause mortality	1.00	0.97 (0.88 to 1.06)	1.07 (0.97 to 1.18)	1.10 (1.01 to 1.23) *	0.02
Cancer mortality	1.00	0.91 (0.78 to 1.07)	0.95 (0.80 to 1.11)	1.08 (0.90 to 1.29)	0.48
CVD mortality	1.00	1.15 (0.99 to 1.33)	1.26 (1.08 to 1.47) **	1.25 (1.05 to 1.49) *	0.004
Other mortality	1.00	0.83 (0.70 to 0.98) *	1.01 (0.85 to 1.20)	0.97 (0.80 to 1.19)	0.74
Meat-based low-carbohydrate-diet score [c]					
All-cause mortality	1.00	0.91 (0.83 to 0.99) *	0.90 (0.82 to 0.98) *	0.87 (0.79 to 0.97) **	0.007
Cancer mortality	1.00	1.04 (0.89 to 1.21)	1.20 (1.03 to 1.40) *	1.08 (0.90 to 1.28)	0.13
CVD mortality	1.00	0.82 (0.71 to 0.94) **	0.75 (0.65 to 0.88) ***	0.78 (0.66 to 0.92) **	0.001
Other mortality	1.00	0.91 (0.77 to 1.07)	0.77 (0.65 to 0.92) **	0.79 (0.65 to 0.96) *	0.003
**Participants with diabetes**					
Overall low- carbohydrate -diet score [a]					
All-cause mortality	1.00	0.90 (0.74 to 1.10)	0.96 (0.79 to 1.17)	1.09 (0.90 to 1.31)	0.25
Cancer mortality	1.00	0.94 (0.65 to 1.37)	0.85 (0.57 to 1.25)	1.00 (0.70 to 1.45)	0.99

CVD mortality	1.00	0.82 (0.61 to 1.11)	0.96 (0.71 to 1.29)	1.14 (0.86 to 1.52)	0.19
Other mortality	1.00	1.00 (0.70 to 1.43)	1.08 (0.76 to 1.55)	1.08 (0.76 to 1.54)	0.59

Continued Table S8

Characteristic	Quartiles of LCD scores				*P* for trend
	1	2	3	4	
Vegetable low-carbohydrate-diet score [b]					
All-cause mortality	1.00	1.00 (0.83 to 1.21)	1.20 (0.99 to 1.46)	1.16 (0.94 to 1.43)	0.06
Cancer mortality	1.00	0.70 (0.49 to 1.01)	0.82 (0.57 to 1.18)	0.82 (0.55 to 1.21)	0.48
CVD mortality	1.00	1.20 (0.88 to 1.62)	1.58 (1.16 to 2.14) **	1.53 (1.10 to 2.12) *	0.003
Other mortality	1.00	1.13 (0.80 to 1.60)	1.21 (0.84 to 1.74)	1.11 (0.75 to 1.64)	0.55
Meat-based low-carbohydrate-diet score [c]					
All-cause mortality	1.00	0.87 (0.72 to 1.05)	0.95 (0.79 to 1.14)	0.99 (0.82 to 1.20)	0.82
Cancer mortality	1.00	0.81 (0.55 to 1.18)	0.98 (0.68 to 1.40)	1.01 (0.70 to 1.46)	0.66
CVD mortality	1.00	0.80 (0.60 to 1.06)	0.96 (0.72 to 1.26)	0.93 (0.70 to 1.23)	0.93
Other mortality	1.00	1.04 (0.74 to 1.46)	0.93 (0.65 to 1.32)	1.09 (0.77 to 1.53)	0.96

[a] Low carbohydrate, high total fat, and high protein intake

[b] Low high-quality carbohydrate, high unsaturated fat, and high plant protein intake

[c] Low low-quality carbohydrate, high saturated fat, and high animal protein intake

[r] All the models adjusted for sex, age, education, family income, smoking, drinking, physical activity, BMI, and history of cancer and CVD.

*:<0.05; **<0.01; ***:<0.001

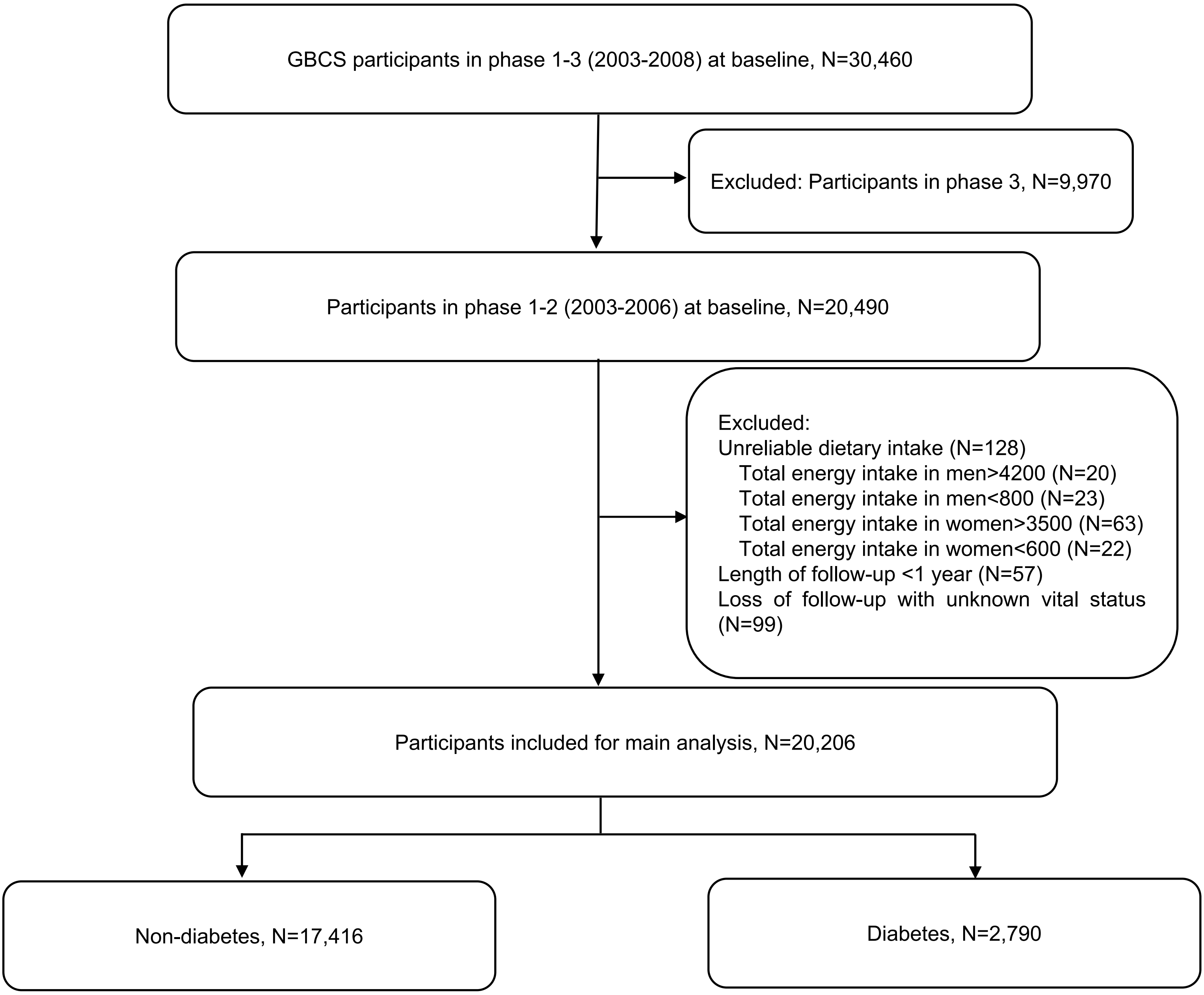

Figure S1. Flowchart showing selection of participants included in the main analysis from Guangzhou Biobank Cohort Study.

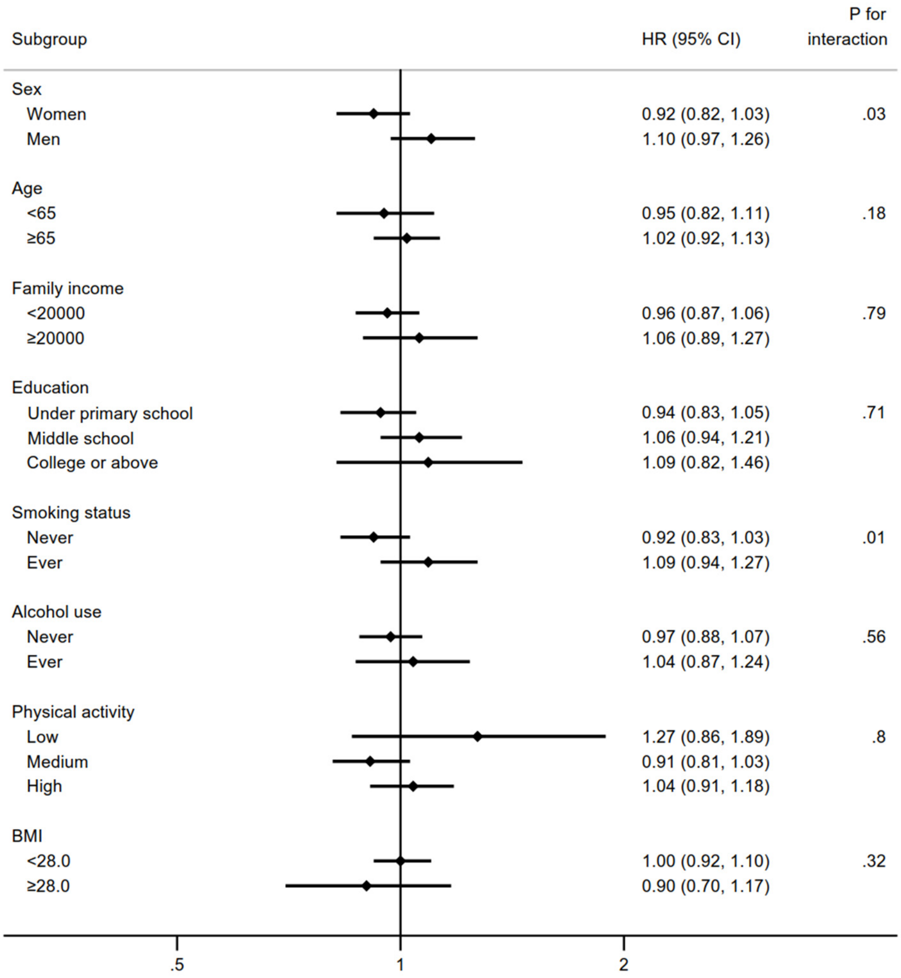

Figure S2. Association of overall LCD score with all-cause mortality by subgroups. All HRs and 95% CIs above were adjusted for sex, age, education, family income, smoking, drinking, physical activity, BMI, and history of cancer and CVD except the corresponding subgroup variates.

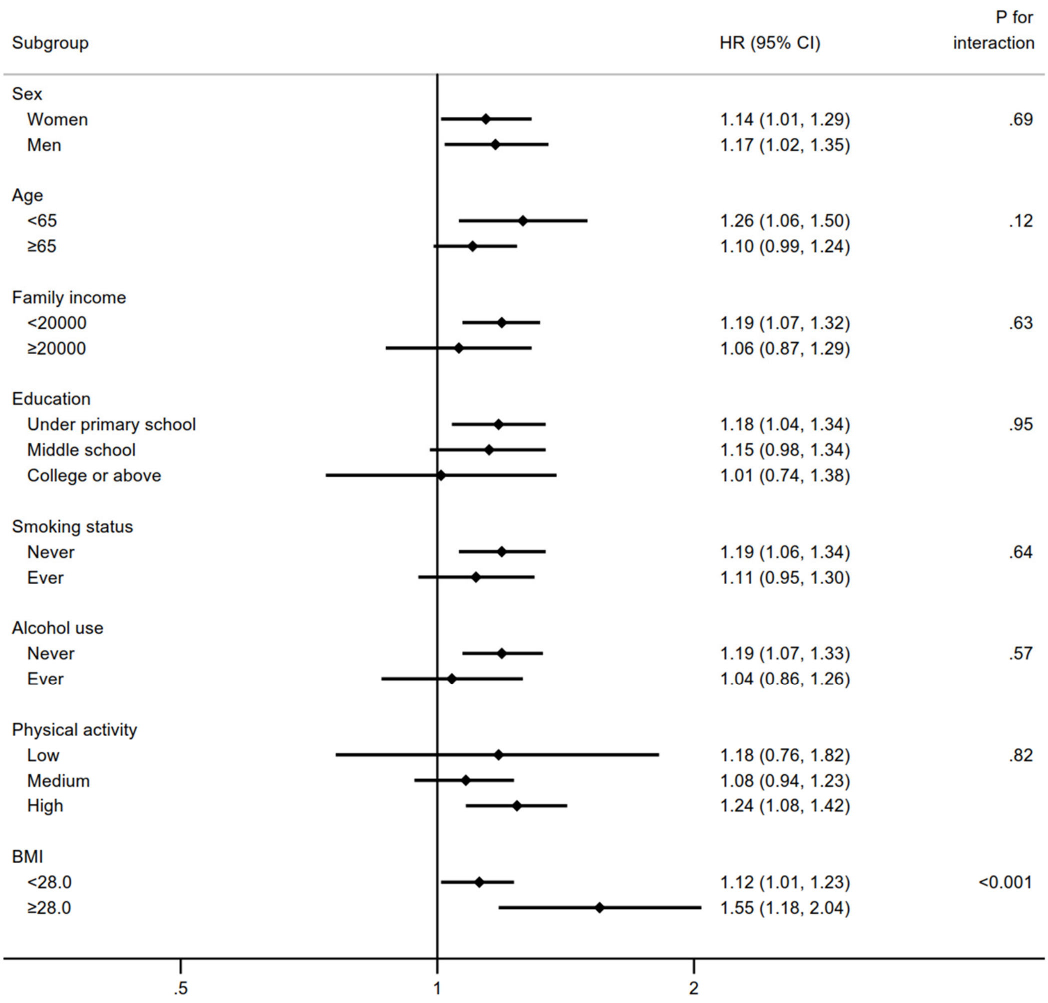

Figure S3. Association of vegetable-based LCD score with all-cause mortality by subgroups. All HRs and 95% CIs above were adjusted for sex, age, education, family income, smoking, drinking, physical activity, BMI, and history of cancer and CVD except the corresponding subgroup variates.

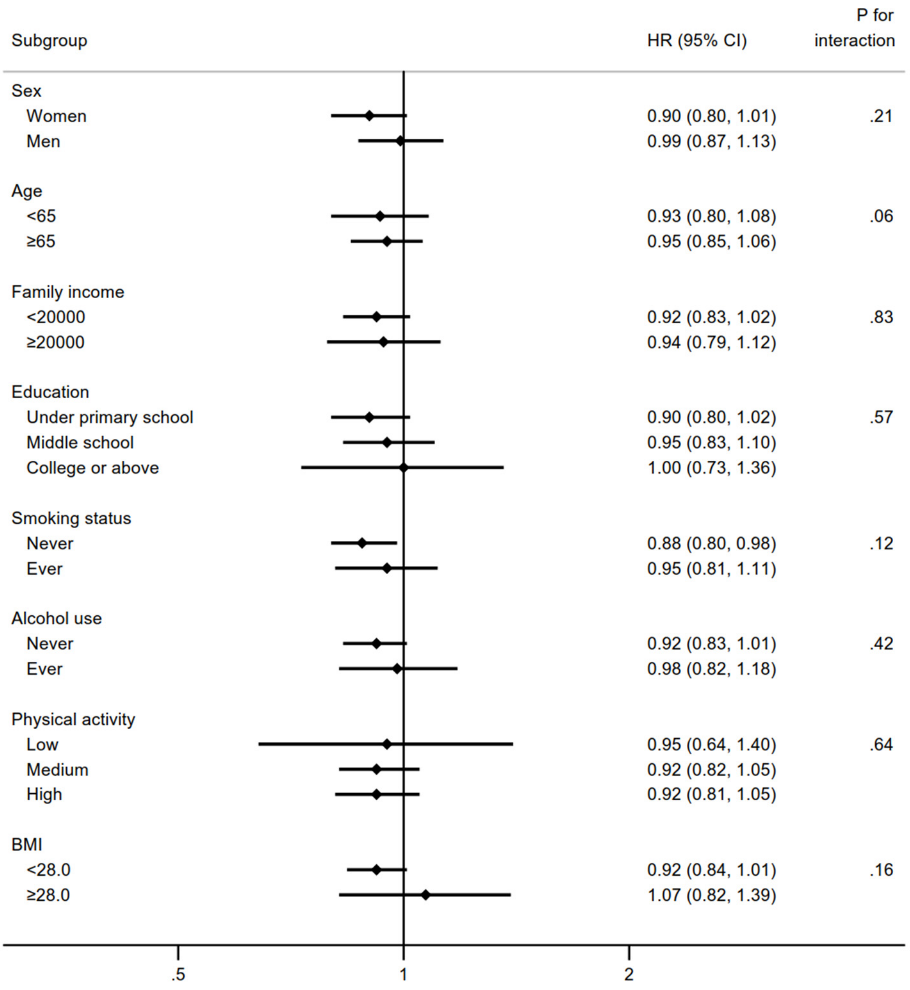

Figure S4. Association of meat-based LCD score with all-cause mortality by subgroups. All HRs and 95% CIs above were adjusted for sex, age, education, family income, smoking, drinking, physical activity, BMI, and history of cancer and CVD except the corresponding subgroup variates.

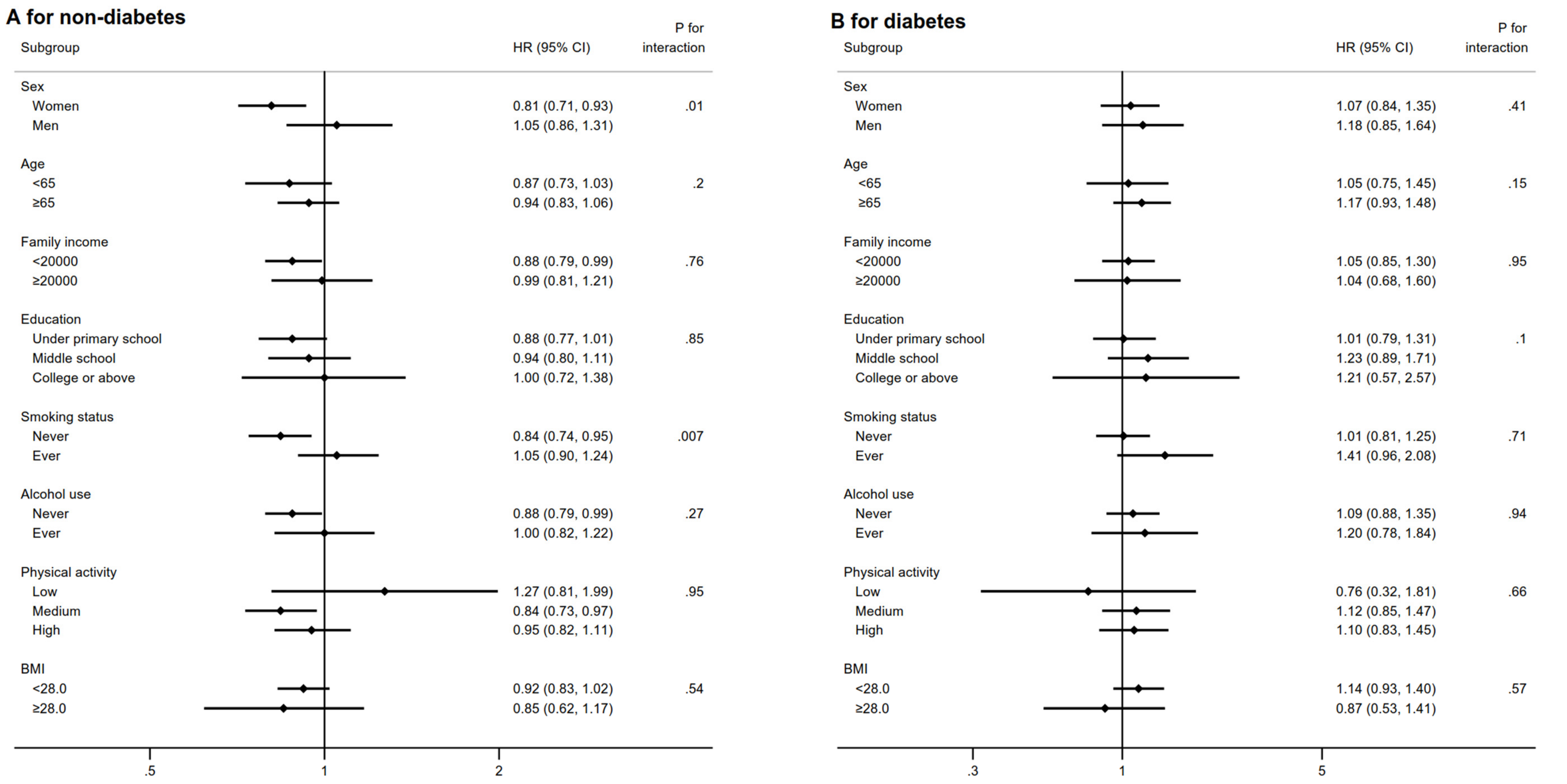

Figure S5. (A) Association of overall LCD score with all-cause mortality in participants without diabetes by subgroup; (B) Association of overall LCD score with all-cause mortality in participants with diabetes by subgroups. All HRs and 95% CIs above were adjusted for sex, age, education, family income, smoking, drinking, physical activity, BMI, and history of cancer and CVD except the corresponding subgroup variates.

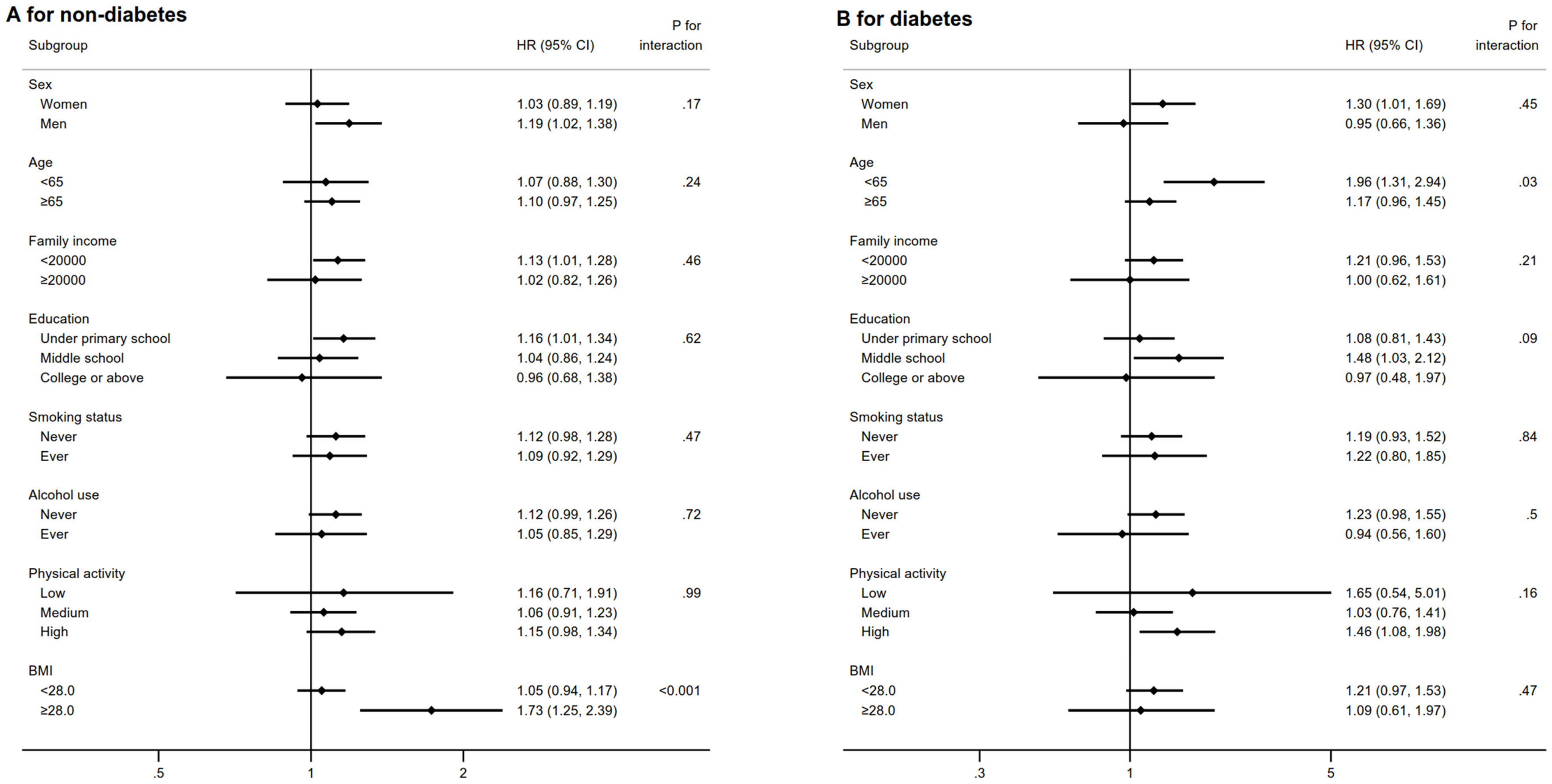

Figure S6. (A) Association of vegetable-based LCD score with all-cause mortality in participants without diabetes by subgroup; (B) Association of vegetable-based LCD score with all-cause mortality in participants with diabetes by subgroup. The HRs and 95%CI above were adjusted for sex, age, education, family income, smoking, drinking, physical activity, BMI, and history of cancer and CVD except the corresponding subgroup variates.

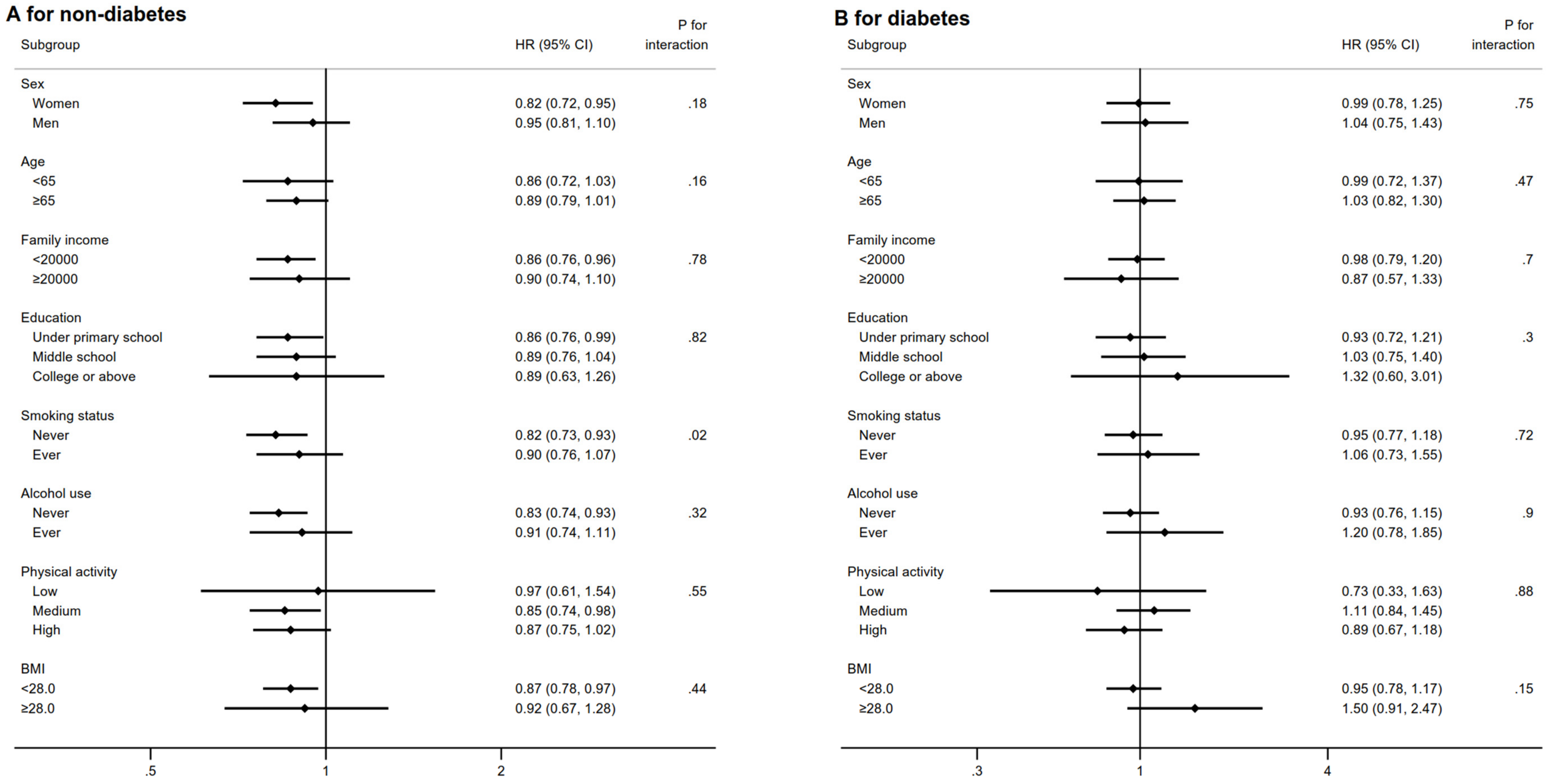

Figure S7. (A) Association of meat-based LCD score with all-cause mortality in participants without diabetes by subgroup; (B) Association of meat-based LCD score with all-cause mortality in participants with diabetes by subgroups. All HRs and 95% CIs above were adjusted for sex, age, education, family income, smoking, drinking, physical activity, BMI, and history of cancer and CVD except the corresponding subgroup variates.

AGReMA[SF]
A Guideline for Reporting Mediation Analyses
Short Form

Table S9. A Guideline for Reporting Mediation Analyses Short-Form (AGReMA-SF) Checklist [a]

Section/Topic	Item Number	Item Description	Reported on page No
**Introduction**			
Objectives	1	State the objectives of the study specific to the mechanisms of interest. The objectives should specify whether the study aims to test or estimate the mechanistic effects	Page 3
**Methods**			
Effects of interest	2	Specify the effects of interest	Page 7
Causal assumptions	3	Specify assumptions about the causal model	Page 8-9
Measurement	4	Clearly describe the interventions or exposures, mediators, outcomes, confounders, and moderators that were used in the analyses. Specify how and when they were measured, the measurement properties, and whether blinded assessment was used	Page 7
Statistical methods	5	Describe the statistical methods used to estimate the causal relationships of interest. This description should specify analytical strategies used to reduce confounding, model building procedures, justification for the inclusion or exclusion of possible interaction terms, modelling assumptions, and methods used to handle missing data. Provide a reference to the statistical software and package used	Page 7-9
**Results**			
Participants	6	Describe baseline characteristics of participants included in mediation analyses. Report the total sample size and number of participants lost during follow-up or with missing data	Page 9-10
Outcomes and estimates	7	Report point estimates and uncertainty estimates for the exposure-mediator and mediator-outcome relationships. If inference concerning the causal relationship of interest is considered feasible given the causal assumptions, report the point estimate and uncertainty estimate	Page11-13
**Discussion**			
Limitations	8	Discuss the limitations of the study including potential sources of bias	Page 17
Interpretation	9	Interpret the estimated effects considering the study's magnitude and uncertainty, plausibility of the causal assumptions, limitations, generalizability of the findings, and results from relevant studies	Page 13-16

From: Lee H, Cashin AG, Lamb SE, Hopewell S, Vansteelandt S, VanderWeele TJ, et al. A Guideline for Reporting Mediation Analyses of Randomized Trials and Observational Studies. The AGReMA Statement. JAMA. 2021;326(11):1045–1056. doi:10.1001/jama.2021.14075

AGReMA-SF is designed for articles that report mediation analyses of randomized trials or observational studies as a secondary focus of a paper. AGReMA-SF should be used in conjunction with CONSORT or STROBE for complete reporting.

For more information, visit: agrema-statement.org

(cc) BY-NC-ND
